# 2-[2-Chloro-5-(trifluoro­methyl)­phen­yl]hexa­hydro­pyrimidine monohydrate

**DOI:** 10.1107/S1600536808027232

**Published:** 2008-08-30

**Authors:** Hoong-Kun Fun, Reza Kia

**Affiliations:** a X-ray Crystallography Unit, School of Physics, Universiti Sains Malaysia, 11800 USM, Penang, Malaysia

## Abstract

The mol­ecule of the title compound, C_11_H_12_ClF_3_N_2_·H_2_O, is a substituted hexa­hydro­pyrimidine. There are two crystallographically independent mol­ecules (*A* and *B*) and two water mol­ecules in the asymmetric unit of the title compound. Inter­molecular C—H⋯Cl (× 2), C—H⋯F, and C—H⋯N (× 2) hydrogen bonds generate *S*(5) ring motifs. The dihedral angle between the two benzene rings is 8.17 (11)°. The F atoms in mol­ecule *B* are disordered over four positions with refined site-occupancies of *ca* 0.35/0.19/0.29/0.17 for the four components. In the crystal structure, mol­ecules are arranged into one-dimensional extended chains along the *c* axis and are further stacked along the *a* axis by directed four-membered O—H⋯O—H inter­actions, forming two-dimensional networks parallel to the *ac* plane. The short distances between the centroids of the benzene rings (3.8002–3.8327 Å) indicate the existence of π–π inter­actions. In addition, the crystal structure is further stabilized by N—H⋯O, O—H⋯N (× 4), N—H⋯Cl and C—H⋯O (× 2) hydrogen-bonding inter­actions.

## Related literature

For bond-length data, see: Allen *et al.* (1987 ▶). For hydrogen-bond motifs, see: Bernstein *et al.* (1995 ▶). For ring conformations, see: Cremer & Pople (1975 ▶). For related literature and properties, see, for example: Riebsomer & Morey (1950 ▶); Finch *et al.* (1952 ▶); Drandarov *et al.* (1999 ▶); Siddiqui *et al.* (1999 ▶); Horvath (1997 ▶); Katritzky *et al.* (2002 ▶).
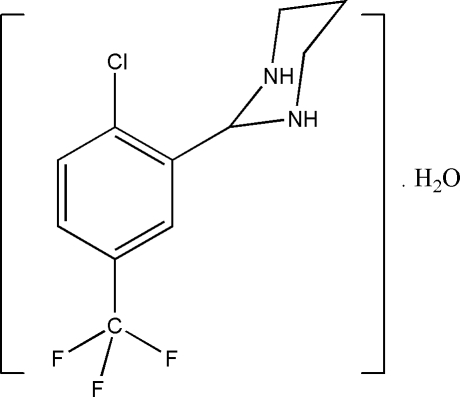


## Experimental

### 

#### Crystal data

C_11_H_12_ClF_3_N_2_·H_2_O
*M* *_r_* = 282.69
Monoclinic, 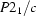

*a* = 7.0745 (2) Å
*b* = 18.6119 (5) Å
*c* = 19.0631 (5) Å
β = 91.010 (2)°
*V* = 2509.65 (12) Å^3^
*Z* = 8
Mo *K*α radiation
μ = 0.33 mm^−1^
*T* = 100.0 (1) K
0.24 × 0.05 × 0.02 mm

#### Data collection

Bruker SMART APEXII CCD area-detector diffractometer
Absorption correction: multi-scan (*SADABS*; Bruker, 2005 ▶) *T*_min_ = 0.924, *T*_max_ = 0.992
30107 measured reflections
7308 independent reflections
4995 reflections with *I* > 2σ(*I*)
*R*_int_ = 0.052

#### Refinement

*R*[*F* ^2^ > 2σ(*F* ^2^)] = 0.064
*wR*(*F* ^2^) = 0.145
*S* = 1.08
7308 reflections
379 parameters
41 restraints
H atoms treated by a mixture of independent and constrained refinement
Δρ_max_ = 0.61 e Å^−3^
Δρ_min_ = −0.60 e Å^−3^

### 

Data collection: *APEX2* (Bruker, 2005 ▶); cell refinement: *APEX2*; data reduction: *SAINT* (Bruker, 2005 ▶); program(s) used to solve structure: *SHELXTL* (Sheldrick, 2008 ▶); program(s) used to refine structure: *SHELXTL*; molecular graphics: *SHELXTL*; software used to prepare material for publication: *SHELXTL* and *PLATON* (Spek, 2003 ▶).

## Supplementary Material

Crystal structure: contains datablocks global, I. DOI: 10.1107/S1600536808027232/at2619sup1.cif

Structure factors: contains datablocks I. DOI: 10.1107/S1600536808027232/at2619Isup2.hkl

Additional supplementary materials:  crystallographic information; 3D view; checkCIF report

## Figures and Tables

**Table 1:** Hydrogen-bond geometry (Å, °)

*D*—H⋯*A*	*D*—H	H⋯*A*	*D*⋯*A*	*D*—H⋯*A*
N1*A*—H1*NA*⋯O1*W*^i^	0.90 (3)	2.07 (4)	2.885 (3)	151 (3)
N1*B*—H1*NB*⋯Cl1*A*^ii^	0.89 (3)	2.76 (3)	3.595 (2)	156 (2)
N2*B*—H2*NB*⋯O2*W*	0.84 (3)	2.18 (4)	2.969 (3)	156 (3)
O1*W*—H1*W*1⋯N2*B*^iii^	0.86 (4)	1.95 (4)	2.815 (3)	174 (4)
O1*W*—H2*W*1⋯N2*A*^iv^	0.87 (4)	2.02 (3)	2.867 (3)	165 (3)
O2*W*—H1*W*2⋯N1*B*^v^	0.83 (3)	2.09 (3)	2.891 (3)	161 (3)
O2*W*—H2*W*2⋯N1*A*^vi^	0.87 (4)	1.98 (4)	2.844 (3)	176 (4)
C2*A*—H2*AA*⋯O2*W*	0.93	2.35	3.249 (3)	164
C5*A*—H5*AA*⋯F1*A*	0.93	2.42	2.747 (3)	100
C5*A*—H5*AA*⋯N1*A*	0.93	2.52	2.835 (3)	100
C7*A*—H7*AA*⋯Cl1*A*	0.98	2.67	3.076 (3)	105
C2*B*—H2*BA*⋯O1*W*^iv^	0.93	2.52	3.414 (3)	162
C5*B*—H5*BA*⋯N2*B*	0.93	2.53	2.838 (3)	100
C7*B*—H7*BA*⋯Cl1*B*	0.98	2.68	3.070 (3)	104

Symmetry codes: (i) 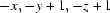; (ii) 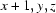; (iii) 
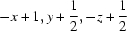
; (iv) 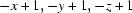; (v) 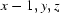; (vi) 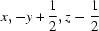.

**Fig. 1.**
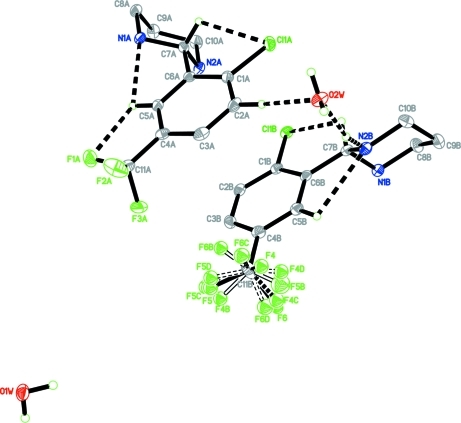
The molecular structure of (I), showing 40% probability displacement ellipsoids. For clarity, only hydrogen atoms of the water molecules and those H atoms involved in intramolecular and intermolecular interactions (shown as dashed lines) were drawn. Open bonds indicate the minor disordered component.

**Fig. 2.**
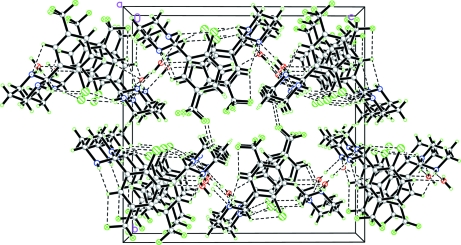
The crystal packing of the major component of (I), viewed down the a-axis, showing 1-D extended chains along the c-axis and stacking of these chains along the a-axis. Intra and intermolecular interactions are shown as dashed lines.

**Fig. 3.**
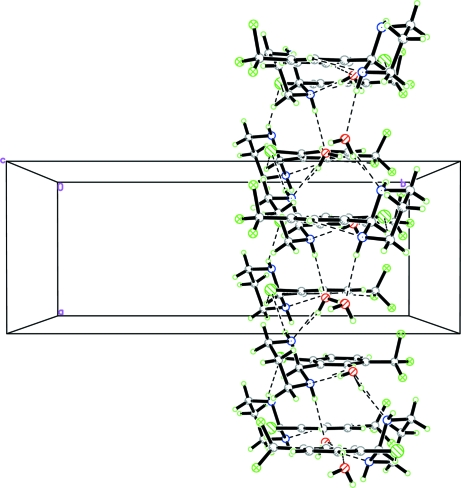
The crystal structure of the major component of (I), showing 1-D extended chains along the a-axis.

## supplementary crystallographic information

### Comment

Hexahydropyrimidines are prepared classically by condensations of substituted propane-1,3-diamines with aldehydes and ketones. Hexahydropyrimidines are biologically important. *N,N'*-Bisalkylhexahydro pyrimidines are effective against Ehrlich carcinoma, LK lymphoma, and Staphylococcus aureus. The hexahydropyrimidine skeleton occurs in alkaloids such as verbamethine and verbametrine. *N*-Substituted hexahydropyrimidines are synthetic intermediates for recently discovered spermidine-nitroimidazole drugs for the treatment of A549 lung carcinoma and structural units in new trypanothione reductase inhibiting ligands for the regulation of oxidative stress in parasitic cells. Benzo-fused hexahydropyrimidines or 1,2,3,4-tetrahydroquinazolines are potential *R*-adrenergic blockers and possess antiplatelet activity.

In the title compound (I) (Fig. 1), intramolecular C—H···Cl (*x* 2), C—H···F, and C—H···N (*x* 2) hydrogen bonds generate *S*(5) ring motifs (Bernstein *et al.*, 1995). The bond lengths and angles are within normal ranges (Allen *et al.*, 1987). There are two crystallographically independent molecules (A, and B) and two water molecules in the asymmetric unit of the title compound. The dihedral angle between the two benzene rings is 8.17 (11)°. The pyrimidine rings in molecules A and B adopt chair conformation with the puckering parameter (Cremer & Pople, 1975) Q=0.603 (3)°; θ=1.2 (3)°; φ=31 (9)° for ring A and Q=0.601 (3)°; θ=2.5 (3)°; φ=48 (6)° for ring B. The CF_3_ fragment in molecule B, was disordered over four positions with the refined site-occupancies of 0.351 (7)/0.189 (5)/0.289 (9)/0.168 (6) for these four components, respectively. In the crystal structure, molecules are arranged into 1-D extended chains along the *c*-axis and are further stacked along the *a*-axis by directed four-membered O—H···O—H interactions to form 2-D networks which is parralell to *ac*-plane. The short distances between the centroids of the benzene rings prove an existence of π-π interactions with distances of 3.8002–3.8327 Å. In addition, the crystal structure is further stabilized by N—H···O, O—H···N (*x* 4), N—H···Cl, and C—H···O (*x* 2) hydrogen bonding interactions.

### Experimental

The title compound was synthesized based on the previous method (Finch *et al.*, 1952). Single crystals suitable for *X*-ray diffraction were obtained by evaporation of an ethanol solution at room temperature.

### Refinement

H atoms bound to the N atoms and water molecules were initially found from the difference Fourier map and refined freely with the parent atoms. The rest of the hydrogen atoms were positioned geometrically and refined as riding model with *U*_iso_(H) = 1.2 *U*_eq_(C). The disordered fluorine atoms of the CF_3_ fragment in molecule B were refined isotropically using C—F bonds distance restraint of 1.300 (5). Their displacement parameters were restrained using rigid bond model.

### Crystal data

**Table:**

C_11_H_12_Cl_1_F_3_N_2_·H_2_O_1_	*F*_000_ = 1168
*M**_r_* = 282.69	*D*_x_ = 1.496 Mg m^−^^3^
Monoclinic, *P*2_1_/*c*	Mo *K*α radiation λ = 0.71073 Å
Hall symbol: -P 2ybc	Cell parameters from 6400 reflections
*a* = 7.0745 (2) Å	θ = 2.4–23.0º
*b* = 18.6119 (5) Å	µ = 0.33 mm^−^^1^
*c* = 19.0631 (5) Å	*T* = 100.0 (1) K
β = 91.010 (2)º	Block, colourless
*V* = 2509.65 (12) Å^3^	0.24 × 0.05 × 0.02 mm
*Z* = 8	

### Data collection

**Table:**

Bruker SMART APEXII CCD area-detector diffractometer	7308 independent reflections
Radiation source: fine-focus sealed tube	4995 reflections with *I* > 2σ(*I*)
Monochromator: graphite	*R*_int_ = 0.053
*T* = 100.0(1) K	θ_max_ = 30.1º
φ and ω scans	θ_min_ = 2.1º
Absorption correction: multi-scan(SADABS; Bruker, 2005)	*h* = −9→9
*T*_min_ = 0.924, *T*_max_ = 0.992	*k* = −26→20
30107 measured reflections	*l* = −16→26

### Refinement

**Table:**

Refinement on *F*^2^	Secondary atom site location: difference Fourier map
Least-squares matrix: full	Hydrogen site location: inferred from neighbouring sites
*R*[*F*^2^ > 2σ(*F*^2^)] = 0.064	H atoms treated by a mixture of independent and constrained refinement
*wR*(*F*^2^) = 0.145	*w* = 1/[σ^2^(*F*_o_^2^) + (0.0411*P*)^2^ + 3.5237*P*] where *P* = (*F*_o_^2^ + 2*F*_c_^2^)/3
*S* = 1.08	(Δ/σ)_max_ < 0.001
7308 reflections	Δρ_max_ = 0.61 e Å^−^^3^
379 parameters	Δρ_min_ = −0.60 e Å^−^^3^
41 restraints	Extinction correction: none
Primary atom site location: structure-invariant direct methods	

### Special details

**Table:**

Experimental. The low-temperature data was collected with the Oxford Cyrosystem Cobra low-temperature attachment.
Geometry. All e.s.d.'s (except the e.s.d. in the dihedral angle between two l.s. planes) are estimated using the full covariance matrix. The cell e.s.d.'s are taken into account individually in the estimation of e.s.d.'s in distances, angles and torsion angles; correlations between e.s.d.'s in cell parameters are only used when they are defined by crystal symmetry. An approximate (isotropic) treatment of cell e.s.d.'s is used for estimating e.s.d.'s involving l.s. planes.
Refinement. Refinement of *F*^2^ against ALL reflections. The weighted *R*-factor *wR* and goodness of fit *S* are based on *F*^2^, conventional *R*-factors *R* are based on *F*, with *F* set to zero for negative *F*^2^. The threshold expression of *F*^2^ > σ(*F*^2^) is used only for calculating *R*-factors(gt) *etc*. and is not relevant to the choice of reflections for refinement. *R*-factors based on *F*^2^ are statistically about twice as large as those based on *F*, and *R*- factors based on ALL data will be even larger.

### Fractional atomic coordinates and isotropic or equivalent isotropic displacement parameters (Å^2^)

**Table:**

	*x*	*y*	*z*	*U*_iso_*/*U*_eq_	Occ. (<1)
Cl1A	0.20441 (9)	0.10260 (4)	0.33890 (4)	0.02426 (16)	
F1A	0.1451 (3)	0.40379 (9)	0.52585 (9)	0.0364 (4)	
F2A	0.0671 (3)	0.44306 (10)	0.42408 (11)	0.0431 (5)	
F3A	0.3579 (2)	0.43149 (10)	0.45284 (10)	0.0382 (5)	
N1A	0.0590 (3)	0.15070 (12)	0.56007 (12)	0.0182 (5)	
N2A	0.3521 (3)	0.10898 (12)	0.51715 (12)	0.0182 (5)	
C1A	0.1928 (3)	0.18840 (15)	0.37603 (14)	0.0195 (5)	
C2A	0.2034 (3)	0.24683 (16)	0.33062 (14)	0.0226 (6)	
H2AA	0.2129	0.2397	0.2825	0.027*	
C3A	0.1998 (3)	0.31528 (16)	0.35780 (14)	0.0227 (6)	
H3AA	0.2056	0.3548	0.3280	0.027*	
C4A	0.1874 (3)	0.32524 (14)	0.42968 (14)	0.0193 (5)	
C5A	0.1752 (3)	0.26648 (14)	0.47436 (14)	0.0169 (5)	
H5AA	0.1667	0.2739	0.5224	0.020*	
C6A	0.1758 (3)	0.19653 (14)	0.44817 (13)	0.0163 (5)	
C7A	0.1603 (3)	0.13238 (14)	0.49649 (13)	0.0176 (5)	
H7AA	0.0946	0.0932	0.4719	0.021*	
C8A	0.0401 (4)	0.08749 (15)	0.60549 (14)	0.0234 (6)	
H8AA	−0.0277	0.0498	0.5805	0.028*	
H8AB	−0.0309	0.1000	0.6468	0.028*	
C9A	0.2355 (4)	0.06120 (16)	0.62705 (15)	0.0246 (6)	
H9AA	0.2249	0.0184	0.6558	0.030*	
H9AB	0.3003	0.0979	0.6545	0.030*	
C10A	0.3474 (4)	0.04425 (15)	0.56176 (15)	0.0240 (6)	
H10A	0.4751	0.0300	0.5749	0.029*	
H10B	0.2883	0.0049	0.5362	0.029*	
C11A	0.1886 (4)	0.40020 (15)	0.45793 (15)	0.0224 (6)	
Cl1B	0.70385 (9)	0.13065 (3)	0.38275 (3)	0.02102 (15)	
C11B	0.6923 (5)	0.43786 (17)	0.29294 (17)	0.0364 (8)	
F4	0.5910 (12)	0.4539 (3)	0.2403 (5)	0.0226 (12)*	0.351 (7)
F5	0.6856 (18)	0.4849 (5)	0.3431 (5)	0.0241 (10)*	0.351 (7)
F6	0.8935 (10)	0.4523 (3)	0.2682 (4)	0.0163 (15)*	0.351 (7)
F4B	0.7521 (16)	0.4863 (4)	0.3361 (5)	0.0226 (12)*	0.189 (5)
F5B	0.716 (2)	0.4505 (6)	0.2289 (7)	0.041 (4)*	0.189 (5)
F6B	0.4747 (12)	0.4605 (4)	0.2919 (6)	0.021 (2)*	0.189 (5)
F4C	0.8220 (14)	0.4554 (3)	0.2541 (5)	0.025 (2)*	0.288 (9)
F5C	0.6978 (15)	0.4818 (5)	0.3569 (5)	0.0241 (10)*	0.288 (9)
F6C	0.5245 (14)	0.4566 (3)	0.2635 (5)	0.027 (2)*	0.288 (9)
F4D	0.639 (2)	0.4462 (6)	0.2155 (8)	0.0226 (12)*	0.167 (6)
F5D	0.619 (2)	0.4824 (6)	0.3297 (7)	0.0241 (10)*	0.167 (6)
F6D	0.866 (2)	0.4612 (8)	0.2826 (8)	0.029 (4)*	0.167 (6)
N1B	0.8534 (3)	0.15394 (12)	0.20979 (12)	0.0178 (5)	
N2B	0.5654 (3)	0.20191 (12)	0.16508 (12)	0.0189 (5)	
C1B	0.6915 (3)	0.21978 (14)	0.35477 (14)	0.0183 (5)	
C2B	0.6998 (3)	0.27234 (15)	0.40623 (14)	0.0200 (5)	
H2BA	0.7062	0.2599	0.4535	0.024*	
C3B	0.6985 (4)	0.34360 (15)	0.38604 (15)	0.0230 (6)	
H3BA	0.7045	0.3797	0.4197	0.028*	
C4B	0.6883 (4)	0.36098 (15)	0.31531 (15)	0.0226 (6)	
C5B	0.6763 (3)	0.30779 (14)	0.26472 (14)	0.0204 (5)	
H5BA	0.6687	0.3205	0.2176	0.024*	
C6B	0.6756 (3)	0.23534 (14)	0.28350 (13)	0.0167 (5)	
C7B	0.6615 (3)	0.17660 (14)	0.22863 (14)	0.0181 (5)	
H7BA	0.5927	0.1355	0.2477	0.022*	
C8B	0.8473 (4)	0.09305 (15)	0.16030 (15)	0.0241 (6)	
H8BA	0.7840	0.0524	0.1814	0.029*	
H8BB	0.9748	0.0785	0.1488	0.029*	
C9B	0.7411 (4)	0.11657 (15)	0.09448 (15)	0.0243 (6)	
H9BA	0.8109	0.1544	0.0714	0.029*	
H9BB	0.7294	0.0764	0.0623	0.029*	
C10B	0.5465 (4)	0.14364 (15)	0.11302 (16)	0.0251 (6)	
H10C	0.4817	0.1613	0.0712	0.030*	
H10D	0.4723	0.1047	0.1322	0.030*	
O1W	0.3328 (3)	0.80736 (12)	0.43314 (11)	0.0258 (5)	
O2W	0.1595 (3)	0.24257 (12)	0.16075 (11)	0.0240 (4)	
H1NA	−0.054 (5)	0.1675 (17)	0.5461 (17)	0.034 (9)*	
H2NA	0.410 (5)	0.0988 (19)	0.478 (2)	0.046 (11)*	
H1NB	0.908 (4)	0.1397 (17)	0.2501 (17)	0.029 (9)*	
H2NB	0.457 (5)	0.2150 (17)	0.1772 (16)	0.028 (8)*	
H1W1	0.370 (5)	0.777 (2)	0.402 (2)	0.053 (12)*	
H2W1	0.440 (5)	0.8277 (19)	0.4430 (18)	0.037 (10)*	
H1W2	0.056 (5)	0.2231 (18)	0.1680 (18)	0.035 (9)*	
H2W2	0.131 (5)	0.277 (2)	0.1315 (19)	0.043 (11)*	

### Atomic displacement parameters (Å^2^)

**Table:**

	*U*^11^	*U*^22^	*U*^33^	*U*^12^	*U*^13^	*U*^23^
Cl1A	0.0223 (3)	0.0306 (4)	0.0199 (3)	0.0021 (3)	−0.0001 (2)	−0.0072 (3)
F1A	0.0535 (11)	0.0211 (9)	0.0352 (11)	0.0018 (8)	0.0155 (9)	−0.0009 (8)
F2A	0.0460 (11)	0.0220 (10)	0.0606 (14)	0.0058 (8)	−0.0230 (10)	0.0078 (9)
F3A	0.0254 (9)	0.0295 (10)	0.0599 (13)	−0.0107 (7)	0.0116 (8)	−0.0090 (9)
N1A	0.0174 (10)	0.0182 (12)	0.0192 (11)	0.0007 (8)	0.0042 (8)	0.0018 (9)
N2A	0.0168 (10)	0.0200 (12)	0.0180 (12)	0.0028 (8)	0.0025 (8)	0.0027 (9)
C1A	0.0153 (11)	0.0243 (14)	0.0188 (13)	−0.0012 (10)	−0.0013 (9)	−0.0022 (11)
C2A	0.0179 (12)	0.0352 (17)	0.0146 (13)	−0.0008 (11)	0.0013 (10)	0.0029 (12)
C3A	0.0185 (12)	0.0286 (16)	0.0209 (14)	−0.0020 (10)	−0.0018 (10)	0.0100 (12)
C4A	0.0142 (11)	0.0205 (14)	0.0232 (14)	−0.0007 (9)	−0.0007 (10)	0.0047 (11)
C5A	0.0152 (11)	0.0205 (14)	0.0150 (12)	−0.0008 (9)	0.0007 (9)	0.0005 (10)
C6A	0.0141 (11)	0.0192 (13)	0.0155 (12)	−0.0020 (9)	−0.0013 (9)	0.0005 (10)
C7A	0.0169 (11)	0.0180 (13)	0.0179 (13)	−0.0004 (9)	0.0000 (9)	−0.0033 (11)
C8A	0.0277 (13)	0.0231 (15)	0.0197 (14)	−0.0025 (11)	0.0076 (11)	0.0024 (11)
C9A	0.0332 (14)	0.0208 (14)	0.0199 (14)	0.0039 (11)	0.0027 (11)	0.0056 (11)
C10A	0.0282 (14)	0.0174 (14)	0.0264 (15)	0.0069 (11)	0.0008 (11)	0.0043 (11)
C11A	0.0185 (12)	0.0246 (15)	0.0242 (15)	−0.0010 (10)	−0.0002 (10)	0.0064 (12)
Cl1B	0.0223 (3)	0.0196 (3)	0.0212 (3)	0.0010 (2)	0.0028 (2)	0.0060 (3)
C11B	0.057 (2)	0.0221 (16)	0.0300 (18)	0.0024 (14)	−0.0009 (15)	−0.0044 (14)
N1B	0.0179 (10)	0.0163 (11)	0.0191 (12)	0.0035 (8)	0.0014 (9)	0.0031 (9)
N2B	0.0137 (10)	0.0225 (12)	0.0204 (12)	0.0015 (8)	0.0005 (8)	0.0015 (9)
C1B	0.0144 (11)	0.0186 (13)	0.0221 (14)	0.0010 (9)	0.0028 (10)	0.0033 (11)
C2B	0.0157 (11)	0.0246 (15)	0.0197 (14)	0.0009 (10)	0.0025 (10)	−0.0001 (11)
C3B	0.0201 (12)	0.0236 (15)	0.0253 (15)	0.0010 (10)	0.0020 (10)	−0.0045 (12)
C4B	0.0202 (12)	0.0185 (14)	0.0293 (15)	0.0012 (10)	0.0018 (11)	0.0016 (12)
C5B	0.0204 (12)	0.0203 (14)	0.0204 (14)	0.0002 (10)	0.0026 (10)	0.0035 (11)
C6B	0.0132 (11)	0.0175 (13)	0.0196 (13)	0.0000 (9)	0.0034 (9)	0.0007 (10)
C7B	0.0165 (11)	0.0182 (13)	0.0199 (13)	−0.0008 (9)	0.0034 (10)	0.0031 (11)
C8B	0.0299 (14)	0.0160 (14)	0.0263 (15)	0.0040 (11)	−0.0001 (11)	−0.0012 (11)
C9B	0.0321 (14)	0.0205 (15)	0.0203 (14)	0.0019 (11)	−0.0006 (11)	−0.0025 (11)
C10B	0.0264 (14)	0.0219 (15)	0.0270 (15)	−0.0039 (11)	−0.0040 (11)	−0.0010 (12)
O1W	0.0178 (9)	0.0318 (12)	0.0279 (11)	0.0006 (8)	0.0029 (8)	−0.0099 (10)
O2W	0.0183 (9)	0.0300 (12)	0.0236 (11)	−0.0010 (8)	0.0003 (8)	0.0074 (9)

### Geometric parameters (Å, °)

**Table:**

Cl1A—C1A	1.749 (3)	C11B—F5	1.298 (10)
F1A—C11A	1.338 (3)	C11B—F6D	1.323 (16)
F2A—C11A	1.331 (3)	C11B—F6C	1.350 (8)
F3A—C11A	1.337 (3)	C11B—F5C	1.467 (10)
N1A—C7A	1.459 (3)	C11B—C4B	1.493 (4)
N1A—C8A	1.468 (3)	C11B—F4D	1.525 (14)
N1A—H1NA	0.90 (3)	C11B—F6	1.532 (7)
N2A—C7A	1.472 (3)	N1B—C7B	1.472 (3)
N2A—C10A	1.475 (3)	N1B—C8B	1.475 (3)
N2A—H2NA	0.88 (4)	N1B—H1NB	0.89 (3)
C1A—C6A	1.391 (4)	N2B—C7B	1.457 (3)
C1A—C2A	1.393 (4)	N2B—C10B	1.475 (4)
C2A—C3A	1.376 (4)	N2B—H2NB	0.84 (3)
C2A—H2AA	0.9300	C1B—C2B	1.386 (4)
C3A—C4A	1.387 (4)	C1B—C6B	1.392 (4)
C3A—H3AA	0.9300	C2B—C3B	1.381 (4)
C4A—C5A	1.390 (4)	C2B—H2BA	0.9300
C4A—C11A	1.495 (4)	C3B—C4B	1.387 (4)
C5A—C6A	1.394 (4)	C3B—H3BA	0.9300
C5A—H5AA	0.9300	C4B—C5B	1.384 (4)
C6A—C7A	1.513 (4)	C5B—C6B	1.395 (4)
C7A—H7AA	0.9800	C5B—H5BA	0.9300
C8A—C9A	1.516 (4)	C6B—C7B	1.515 (4)
C8A—H8AA	0.9700	C7B—H7BA	0.9800
C8A—H8AB	0.9700	C8B—C9B	1.516 (4)
C9A—C10A	1.520 (4)	C8B—H8BA	0.9700
C9A—H9AA	0.9700	C8B—H8BB	0.9700
C9A—H9AB	0.9700	C9B—C10B	1.514 (4)
C10A—H10A	0.9700	C9B—H9BA	0.9700
C10A—H10B	0.9700	C9B—H9BB	0.9700
Cl1B—C1B	1.744 (3)	C10B—H10C	0.9700
C11B—F5D	1.209 (11)	C10B—H10D	0.9700
C11B—F4C	1.233 (8)	O1W—H1W1	0.86 (4)
C11B—F5B	1.257 (13)	O1W—H2W1	0.87 (4)
C11B—F4	1.258 (6)	O2W—H1W2	0.83 (4)
C11B—F4B	1.287 (5)	O2W—H2W2	0.87 (4)
			
C7A—N1A—C8A	110.7 (2)	F4—C11B—F5C	122.5 (6)
C7A—N1A—H1NA	107 (2)	F4B—C11B—F5C	22.3 (6)
C8A—N1A—H1NA	111 (2)	F5—C11B—F5C	9.3 (7)
C7A—N2A—C10A	111.5 (2)	F6D—C11B—F5C	86.0 (9)
C7A—N2A—H2NA	106 (2)	F6C—C11B—F5C	102.2 (6)
C10A—N2A—H2NA	109 (2)	F5D—C11B—C4B	118.8 (6)
C6A—C1A—C2A	122.4 (2)	F4C—C11B—C4B	116.3 (4)
C6A—C1A—Cl1A	120.3 (2)	F5B—C11B—C4B	117.4 (6)
C2A—C1A—Cl1A	117.2 (2)	F4—C11B—C4B	116.2 (3)
C3A—C2A—C1A	119.2 (2)	F4B—C11B—C4B	119.7 (5)
C3A—C2A—H2AA	120.4	F5—C11B—C4B	115.7 (5)
C1A—C2A—H2AA	120.4	F6D—C11B—C4B	112.3 (7)
C2A—C3A—C4A	119.8 (3)	F6C—C11B—C4B	110.3 (4)
C2A—C3A—H3AA	120.1	F5C—C11B—C4B	107.3 (5)
C4A—C3A—H3AA	120.1	F5D—C11B—F4D	113.0 (8)
C3A—C4A—C5A	120.4 (3)	F4C—C11B—F4D	64.3 (7)
C3A—C4A—C11A	118.7 (2)	F5B—C11B—F4D	22.4 (7)
C5A—C4A—C11A	120.9 (2)	F4—C11B—F4D	22.6 (5)
C4A—C5A—C6A	121.0 (2)	F4B—C11B—F4D	128.4 (7)
C4A—C5A—H5AA	119.5	F5—C11B—F4D	129.3 (6)
C6A—C5A—H5AA	119.5	F6D—C11B—F4D	92.1 (8)
C1A—C6A—C5A	117.2 (2)	F6C—C11B—F4D	51.0 (7)
C1A—C6A—C7A	121.6 (2)	F5C—C11B—F4D	138.6 (6)
C5A—C6A—C7A	121.2 (2)	C4B—C11B—F4D	111.6 (5)
N1A—C7A—N2A	108.1 (2)	F5D—C11B—F6	118.0 (7)
N1A—C7A—C6A	111.3 (2)	F4C—C11B—F6	20.4 (5)
N2A—C7A—C6A	108.7 (2)	F5B—C11B—F6	61.9 (8)
N1A—C7A—H7AA	109.6	F4—C11B—F6	103.4 (5)
N2A—C7A—H7AA	109.6	F4B—C11B—F6	77.1 (6)
C6A—C7A—H7AA	109.6	F5—C11B—F6	98.9 (6)
N1A—C8A—C9A	109.0 (2)	F6D—C11B—F6	12.7 (7)
N1A—C8A—H8AA	109.9	F6C—C11B—F6	129.9 (6)
C9A—C8A—H8AA	109.9	F5C—C11B—F6	98.4 (6)
N1A—C8A—H8AB	109.9	C4B—C11B—F6	106.2 (3)
C9A—C8A—H8AB	109.9	F4D—C11B—F6	84.2 (6)
H8AA—C8A—H8AB	108.3	C7B—N1B—C8B	111.1 (2)
C8A—C9A—C10A	109.3 (2)	C7B—N1B—H1NB	105 (2)
C8A—C9A—H9AA	109.8	C8B—N1B—H1NB	109 (2)
C10A—C9A—H9AA	109.8	C7B—N2B—C10B	110.9 (2)
C8A—C9A—H9AB	109.8	C7B—N2B—H2NB	106 (2)
C10A—C9A—H9AB	109.8	C10B—N2B—H2NB	109 (2)
H9AA—C9A—H9AB	108.3	C2B—C1B—C6B	123.1 (2)
N2A—C10A—C9A	108.6 (2)	C2B—C1B—Cl1B	117.0 (2)
N2A—C10A—H10A	110.0	C6B—C1B—Cl1B	119.9 (2)
C9A—C10A—H10A	110.0	C3B—C2B—C1B	118.7 (3)
N2A—C10A—H10B	110.0	C3B—C2B—H2BA	120.6
C9A—C10A—H10B	110.0	C1B—C2B—H2BA	120.6
H10A—C10A—H10B	108.3	C2B—C3B—C4B	119.7 (3)
F2A—C11A—F3A	106.0 (2)	C2B—C3B—H3BA	120.2
F2A—C11A—F1A	106.4 (2)	C4B—C3B—H3BA	120.2
F3A—C11A—F1A	105.7 (2)	C5B—C4B—C3B	120.8 (3)
F2A—C11A—C4A	112.6 (2)	C5B—C4B—C11B	119.2 (3)
F3A—C11A—C4A	112.3 (2)	C3B—C4B—C11B	120.0 (3)
F1A—C11A—C4A	113.3 (2)	C4B—C5B—C6B	120.9 (3)
F5D—C11B—F4C	119.8 (7)	C4B—C5B—H5BA	119.6
F5D—C11B—F5B	120.0 (8)	C6B—C5B—H5BA	119.6
F4C—C11B—F5B	41.9 (7)	C1B—C6B—C5B	116.8 (2)
F5D—C11B—F4	93.2 (7)	C1B—C6B—C7B	121.8 (2)
F4C—C11B—F4	83.0 (5)	C5B—C6B—C7B	121.4 (2)
F5B—C11B—F4	42.6 (8)	N2B—C7B—N1B	108.0 (2)
F5D—C11B—F4B	44.6 (8)	N2B—C7B—C6B	111.4 (2)
F4C—C11B—F4B	87.6 (7)	N1B—C7B—C6B	109.0 (2)
F5B—C11B—F4B	116.3 (8)	N2B—C7B—H7BA	109.5
F4—C11B—F4B	121.4 (6)	N1B—C7B—H7BA	109.5
F5D—C11B—F5	24.3 (6)	C6B—C7B—H7BA	109.5
F4C—C11B—F5	107.5 (6)	N1B—C8B—C9B	108.4 (2)
F5B—C11B—F5	126.7 (7)	N1B—C8B—H8BA	110.0
F4—C11B—F5	113.6 (5)	C9B—C8B—H8BA	110.0
F4B—C11B—F5	21.9 (6)	N1B—C8B—H8BB	110.0
F5D—C11B—F6D	105.7 (9)	C9B—C8B—H8BB	110.0
F4C—C11B—F6D	28.3 (7)	H8BA—C8B—H8BB	108.4
F5B—C11B—F6D	69.8 (10)	C10B—C9B—C8B	110.0 (2)
F4—C11B—F6D	108.7 (8)	C10B—C9B—H9BA	109.7
F4B—C11B—F6D	64.5 (8)	C8B—C9B—H9BA	109.7
F5—C11B—F6D	86.2 (8)	C10B—C9B—H9BB	109.7
F5D—C11B—F6C	71.4 (8)	C8B—C9B—H9BB	109.7
F4C—C11B—F6C	109.8 (6)	H9BA—C9B—H9BB	108.2
F5B—C11B—F6C	71.2 (8)	N2B—C10B—C9B	109.2 (2)
F4—C11B—F6C	28.7 (4)	N2B—C10B—H10C	109.8
F4B—C11B—F6C	111.2 (6)	C9B—C10B—H10C	109.8
F5—C11B—F6C	95.1 (6)	N2B—C10B—H10D	109.8
F6D—C11B—F6C	131.7 (8)	C9B—C10B—H10D	109.8
F5D—C11B—F5C	30.9 (8)	H10C—C10B—H10D	108.3
F4C—C11B—F5C	110.0 (7)	H1W1—O1W—H2W1	99 (3)
F5B—C11B—F5C	134.4 (7)	H1W2—O2W—H2W2	104 (3)
			
C6A—C1A—C2A—C3A	0.9 (4)	F4—C11B—C4B—C5B	36.6 (7)
Cl1A—C1A—C2A—C3A	−178.32 (19)	F4B—C11B—C4B—C5B	−161.8 (7)
C1A—C2A—C3A—C4A	0.6 (4)	F5—C11B—C4B—C5B	173.8 (7)
C2A—C3A—C4A—C5A	−1.1 (4)	F6D—C11B—C4B—C5B	−89.4 (8)
C2A—C3A—C4A—C11A	178.5 (2)	F6C—C11B—C4B—C5B	67.3 (6)
C3A—C4A—C5A—C6A	0.2 (4)	F5C—C11B—C4B—C5B	177.8 (5)
C11A—C4A—C5A—C6A	−179.4 (2)	F4D—C11B—C4B—C5B	12.4 (7)
C2A—C1A—C6A—C5A	−1.9 (4)	F6—C11B—C4B—C5B	−77.7 (4)
Cl1A—C1A—C6A—C5A	177.39 (18)	F5D—C11B—C4B—C3B	−34.1 (9)
C2A—C1A—C6A—C7A	178.7 (2)	F4C—C11B—C4B—C3B	120.7 (6)
Cl1A—C1A—C6A—C7A	−2.0 (3)	F5B—C11B—C4B—C3B	168.0 (8)
C4A—C5A—C6A—C1A	1.3 (3)	F4—C11B—C4B—C3B	−144.0 (6)
C4A—C5A—C6A—C7A	−179.3 (2)	F4B—C11B—C4B—C3B	17.5 (7)
C8A—N1A—C7A—N2A	−62.4 (3)	F5—C11B—C4B—C3B	−6.9 (8)
C8A—N1A—C7A—C6A	178.3 (2)	F6D—C11B—C4B—C3B	89.9 (8)
C10A—N2A—C7A—N1A	61.8 (3)	F6C—C11B—C4B—C3B	−113.4 (6)
C10A—N2A—C7A—C6A	−177.3 (2)	F5C—C11B—C4B—C3B	−2.8 (6)
C1A—C6A—C7A—N1A	−153.3 (2)	F4D—C11B—C4B—C3B	−168.2 (7)
C5A—C6A—C7A—N1A	27.3 (3)	F6—C11B—C4B—C3B	101.7 (4)
C1A—C6A—C7A—N2A	87.8 (3)	C3B—C4B—C5B—C6B	−0.5 (4)
C5A—C6A—C7A—N2A	−91.6 (3)	C11B—C4B—C5B—C6B	178.9 (3)
C7A—N1A—C8A—C9A	61.3 (3)	C2B—C1B—C6B—C5B	2.7 (4)
N1A—C8A—C9A—C10A	−57.6 (3)	Cl1B—C1B—C6B—C5B	−176.75 (18)
C7A—N2A—C10A—C9A	−59.3 (3)	C2B—C1B—C6B—C7B	−178.4 (2)
C8A—C9A—C10A—N2A	56.3 (3)	Cl1B—C1B—C6B—C7B	2.2 (3)
C3A—C4A—C11A—F2A	48.9 (3)	C4B—C5B—C6B—C1B	−1.3 (4)
C5A—C4A—C11A—F2A	−131.5 (3)	C4B—C5B—C6B—C7B	179.7 (2)
C3A—C4A—C11A—F3A	−70.7 (3)	C10B—N2B—C7B—N1B	62.4 (3)
C5A—C4A—C11A—F3A	108.9 (3)	C10B—N2B—C7B—C6B	−178.0 (2)
C3A—C4A—C11A—F1A	169.7 (2)	C8B—N1B—C7B—N2B	−63.0 (3)
C5A—C4A—C11A—F1A	−10.7 (3)	C8B—N1B—C7B—C6B	175.8 (2)
C6B—C1B—C2B—C3B	−2.1 (4)	C1B—C6B—C7B—N2B	154.7 (2)
Cl1B—C1B—C2B—C3B	177.29 (19)	C5B—C6B—C7B—N2B	−26.4 (3)
C1B—C2B—C3B—C4B	0.2 (4)	C1B—C6B—C7B—N1B	−86.3 (3)
C2B—C3B—C4B—C5B	1.0 (4)	C5B—C6B—C7B—N1B	92.6 (3)
C2B—C3B—C4B—C11B	−178.3 (3)	C7B—N1B—C8B—C9B	60.2 (3)
F5D—C11B—C4B—C5B	146.6 (9)	N1B—C8B—C9B—C10B	−56.1 (3)
F4C—C11B—C4B—C5B	−58.6 (7)	C7B—N2B—C10B—C9B	−59.7 (3)
F5B—C11B—C4B—C5B	−11.4 (9)	C8B—C9B—C10B—N2B	56.0 (3)

### Hydrogen-bond geometry (Å, °)

**Table:**

*D*—H···*A*	*D*—H	H···*A*	*D*···*A*	*D*—H···*A*
N1A—H1NA···O1W^i^	0.90 (3)	2.07 (4)	2.885 (3)	151 (3)
N1B—H1NB···Cl1A^ii^	0.89 (3)	2.76 (3)	3.595 (2)	156 (2)
N2B—H2NB···O2W	0.84 (3)	2.18 (4)	2.969 (3)	156 (3)
O1W—H1W1···N2B^iii^	0.86 (4)	1.95 (4)	2.815 (3)	174 (4)
O1W—H2W1···N2A^iv^	0.87 (4)	2.02 (3)	2.867 (3)	165 (3)
O2W—H1W2···N1B^v^	0.83 (3)	2.09 (3)	2.891 (3)	161 (3)
O2W—H2W2···N1A^vi^	0.87 (4)	1.98 (4)	2.844 (3)	176 (4)
C2A—H2AA···O2W	0.93	2.35	3.249 (3)	164
C5A—H5AA···F1A	0.93	2.42	2.747 (3)	100
C5A—H5AA···N1A	0.93	2.52	2.835 (3)	100
C7A—H7AA···Cl1A	0.98	2.67	3.076 (3)	105
C2B—H2BA···O1W^iv^	0.93	2.52	3.414 (3)	162
C5B—H5BA···N2B	0.93	2.53	2.838 (3)	100
C7B—H7BA···Cl1B	0.98	2.68	3.070 (3)	104

Symmetry codes: (i) −*x*, −*y*+1, −*z*+1; (ii) *x*+1, *y*, *z*; (iii) −*x*+1, *y*+1/2, −*z*+1/2; (iv) −*x*+1, −*y*+1, −*z*+1; (v) *x*−1, *y*, *z*; (vi) *x*, −*y*+1/2, *z*−1/2.
